# Decreased Mitochondrial DNA Content in Association with Exposure to Polycyclic Aromatic Hydrocarbons in House Dust during Wintertime: From a Population Enquiry to Cell Culture

**DOI:** 10.1371/journal.pone.0063208

**Published:** 2013-05-03

**Authors:** Nicky Pieters, Gudrun Koppen, Karen Smeets, Dorota Napierska, Michelle Plusquin, Sofie De Prins, Hendrik Van De Weghe, Vera Nelen, Bianca Cox, Ann Cuypers, Peter Hoet, Greet Schoeters, Tim S. Nawrot

**Affiliations:** 1 Centre for Environmental Sciences, Hasselt University, Diepenbeek, Belgium; 2 Environmental Risk & Health Unit, VITO (Flemish Institute of Technological Research), Mol, Belgium; 3 Department of Public Health & Primary Care, Occupational & Environmental Medicine, Leuven University (KU Leuven), Leuven, Belgium; 4 Faculty of Pharmaceutical, Biomedical and Veterinary Sciences, University of Antwerp, Antwerp, Belgium; 5 Environment and Health Unit, Provincial Institute of Hygiene, Antwerp, Belgium; Universidad Pablo de Olavide, Centro Andaluz de Biología del Desarrollo-CSIC, Spain

## Abstract

Polycyclic aromatic hydrocarbons (PAHs) are widespread environmental pollutants that are formed in combustion processes. At the cellular level, exposure to PAHs causes oxidative stress and/or some of it congeners bind to DNA, which may interact with mitochondrial function. However, the influence of these pollutants on mitochondrial DNA (mtDNA) content remains largely unknown. We determined whether indoor exposure to PAHs is associated with mitochondrial damage as represented by blood mtDNA content. Blood mtDNA content (ratio mitochondrial/nuclear DNA copy number) was determined by real-time qPCR in 46 persons, both in winter and summer. Indoor PAH exposure was estimated by measuring PAHs in sedimented house dust, including 6 volatile PAHs and 8 non-volatile PAHs. Biomarkers of oxidative stress at the level of DNA and lipid peroxidation were measured. In addition to the epidemiologic enquiry, we exposed human TK6 cells during 24 h at various concentrations (range: 0 to 500 µM) of benzo*(a)*pyrene and determined mtDNA content. Mean blood mtDNA content averaged (±SD) 0.95±0.185. The median PAH content amounted 554.1 ng/g dust (25^th^–75^th^ percentile: 390.7–767.3) and 1385****ng/g dust (25^th^–75^th^ percentile: 1000–1980) in winter for volatile and non-volatile PAHs respectively. Independent for gender, age, BMI and the consumption of grilled meat or fish, blood mtDNA content decreased by 9.85% (95% CI: −15.16 to −4.2; p = 0.002) for each doubling of non-volatile PAH content in the house dust in winter. The corresponding estimate for volatile PAHs was −7.3% (95% CI: −13.71 to −0.42; p = 0.04). Measurements of oxidative stress were not correlated with PAH exposure. During summer months no association was found between mtDNA content and PAH concentration. The ability of benzo(*a*)pyrene (range 0 µM to 500 µM) to lower mtDNA content was confirmed *in vitro* in human TK6 cells. Based on these findings, mtDNA content can be a target of PAH toxicity in humans.

## Introduction

Polycyclic aromatic hydrocarbons (PAHs) are widespread pollutants, which are formed during incomplete combustion processes. Important sources of PAH exposure are motorized traffic and heating with fossil fuels. Some of the reactive metabolites of PAHs can bind to and damage macromolecules, including DNA. PAHs can induce oxidative stress indirectly trough cytochrome P450, epoxide hydrolase and dihydriodiol dehydrogenase, which results in the generation of quinones [1]. These redox active quinones are able to produce reactive oxygen species (ROS), thereby causing oxidative stress. It was shown that the PAHs and quinones, present on ultrafine particles, lead to functional and structural damage of the mitochondria, such as decreases in the mitochondrial membrane potential, either direct or secondary through oxidative damage [2].

In normal conditions, ROS are generated in the mitochondria as metabolic by-products of the aerobic mechanism. ROS are continuously produced at the level of the mitochondrial electron transfer chain, where superoxide is produced by the one-electron reduction of oxygen [3]. Each mammalian cell contains approximately 200 to 2000 mitochondria, each carrying 2 to 10 copies of mitochondrial DNA. The mitochondrial DNA copy number is correlated with the amount and size of mitochondria [4]. Compared with nuclear DNA, mitochondrial DNA is more susceptible to damage because it lacks protective histones and has a diminished DNA repair capacity. As a result mitochondrial DNA has a high mutation rate and is particularly vulnerable to ROS-induced damage [5], [6], as well as to damage directly by adducts [7]. Initially, cells challenged with ROS synthesize more copies of their mitochondrial DNA and increase the number of mitochondria to compensate for the damage, resulting in a vicious circle of more ROS production from damaged mitochondria. However, in time, as defective mitochondria accumulate, bio-energetic and replicative function declines, leading to decreased or no synthesis of mitochondrial DNA [8].

Surrogates of indoor PAH exposure have been measured in several environmental media, including air [9], [10], [11] and house dust [12], [13], [14], [15], [16], [17], [18], [19], [20]. Because PAHs can accumulate in carpets over years and decades, house dust PAH concentrations may be long-term predictors of indoor PAH exposure. According to Gevao et al. 2007, inadvertent dust ingestion is responsible for 11% of non-dietary total PAH exposure in adults and as much as 42% in young children [19].

In the present study, we investigate the association of blood mitochondrial DNA content in association with indoor exposure to different PAH congeners. To establish a higher level of causality we performed, in addition to our study in humans, an *in vitro* experiment in which human cells were exposed to different concentrations of benzo*(a)*pyrene.

## Results

### Population Study

#### Characteristics of the study population

The median age of the 46 participants was 40 years (IQR: 32–47). Fifty two percent were men (table 1). Ten persons (22%) were former smokers. The participants had a mean (± SD) body mass index (BMI) of 24.2 kg/m^2^ (±3.3) in winter and 23.8 kg/m^2^ (±3.4) in summer (p = 0.65). The mean relative mitochondrial DNA content was similar for both seasons and amounted 0.954 in winter and 0.947 in summer (p = 0.85, table 2).

**Table 1:** Characteristics of the study population.

Characteristics		median (IQR) or number (%)
Male		24 (52.2%)
Age (y)		40 (32;47)
Former smokers		10 (22%)
Heating source		
	Central heating	21 (84%)
	Electricity	4 (16%)
	Woodstove	8 (35%)

Data are presented as median (IQR = 25–75 percentile)or number (%).

Heating source data per household.

**Table 2:** Characteristics of the study population stratified for winter and summer.

Characteristics	Mean ± SD or number (%)		
	Winter	Summer	p-value
BMI	24.2±3.3	23.8±3.4	0.65
Use of medication			
Bronchospasmolytica	3 (6.5%)	2 (4.4%)	0.99
H1 histamine antagonist	2 (4.4%)	2 (4.4%)	0.99
Glucocorticoïds	1 (2.2%)	0	0.99
Antihypertensives	2 (4.4%)	2 (4.4%)	0.99
Frequency consumption of grilled food*			
Daily	0	0	0.99
Weekly	2 (4.4%)	7 (15.2%)	0.16
Monthly	2 (4.4%)	20 (43.5%)	0.0001
Relative mitochondrial DNA content (mtDNAcn/nDNAcn)	0.954±0.18	0.947±0.19	0.85

Data are presented as number (%) or arithmetic mean ±SD.

* Frequency of consumption of grilled food: data for one person, winter missing.

#### Exposure levels to PAHs

**Figure 1:**
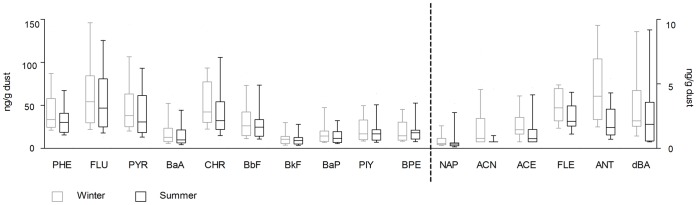
Median amount of each PAH-component in winter and in summer (ng/g dust). PHE, phenanthrene; FLU, fluoranthene; PYR, pyrene; BaA, benzo*(a)*anthracene; CHR, chrysene; BbF, benzo*(b)*fluoranthene; BkF, benzo(*k)*fluoranthene; BAP, benzo*(a)*pyrene; PIY, indenol*(1,2,3-cd)*pyrene; BPE, benzo*(g,h,i)*perylene; NAP, naphthalene; ACN, acenaphtylene; ACE, acenapthnene; FLE, fluorene; ANT, anthracene; dBA, dibenzo*(a,h)*anthracene.

The non-volatile PAH en volatile PAH concentrations in house dust were higher in the winter period than in summer (table 3). The 3- and 4-ring phenanthrene, fluorene, pyrene (resp. ca. 12%, 17% and 12%) and the 4- and 5-ring structures chrysene and benzo*[b]*fluoranthene (resp. ca. 14% and 10%), made up the most important contribution to the measured PAH concentration in house dust (Figure 1). Sixteen participants (35%) lived in a house with regular use of a woodstove in winter. The volatile PAH, non-volatile PAH and benzo*(a)*pyrene concentration tended to be higher, although not significantly, in houses with this heating device compared to houses with another heating source in winter (data not shown).

**Table 3:** Median amount (25th–75th percentile) of volatile and non-volatile PAHs and benzo*(a)*pyrene found in house dust (ng/g dust).

	Winter			Summer			
PAH	Median	25th P	75th P	Median	25th P	75th P	p-value
Volatile	554	390	767	446	311	655	0.04
Non-volatile	1385	1000	1980	1258	733	1762	0.05
Benzo*(a)*pyrene	144	85	180	116	66	206	0.11

#### Relative mitochondrial DNA content and indoor exposure to PAHs

**Figure 2:**
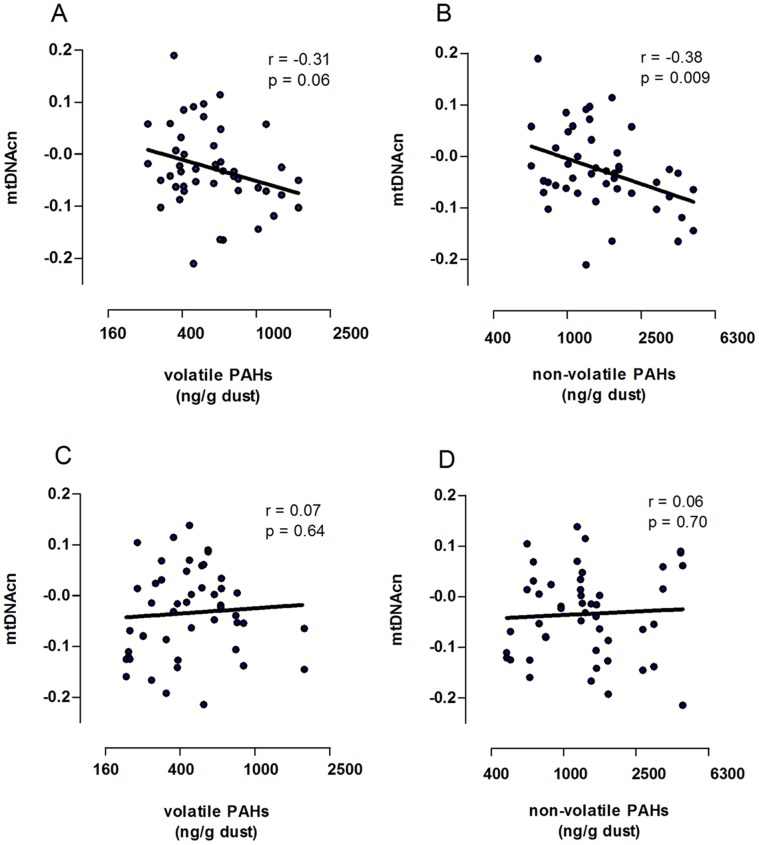
Association between mitochondrial DNA content and PAH exposure in winter and in summer. Four correlation plots are given, each indicating different PAH exposure, volatile PAHs in house dust in winter (A), non-volatile PAHs in house dust in winter (B), volatile PAHs in house dust in summer in (C) and non-volatile PAHs in house dust in summer in (D). Values of mitochondrial DNA content (mtDNAcn) are log transformed.

Blood mitochondrial DNA content was similar in men and women (0.96 vs 0.99, p = 0.48). We noticed significant season-by-PAH exposure interactions on mitochondrial DNA content. Therefore, we analyzed the data for summer and winter separately. In winter, both before (figure 2) and after (table 4) cumulative adjustment for gender, age, BMI and the consumption of grilled meat or fish, blood mitochondrial DNA content was inversely and independently correlated with the indoor PAH dust concentration. When the analysis was repeated separately for non-volatile and volatile PAHs, we found that the effect was mostly attributed to non-volatile PAHs (table 4). We found a decrease of 9.85% (95% CI: −15.16% to −4.2%, p = 0.002) in mitochondrial DNA content for each doubling in non-volatile PAH concentration and a 7.3% decrease (95% CI: −13.71% to −0.42%, p = 0.04) for a doubling in volatile PAH, when adjusted for aforementioned variables. In addition we ran a separate multivariate analysis using benzo(*a*)pyrene dust exposure. Each doubling in benzo(*a*)pyrene exposuse was associated with a 7.18% decrease (95%CI: −11.82% to −2.3%, p = 0.007) in mitochondrial DNA content. In summer, with adjustments applied as before, blood mitochondrial DNA content was not associated with indoor PAH.

**Table 4:** Estimated change (95% CI) in mitochondrial DNA (mtDNA) content in association with PAHs exposure.

	Winter			Summer		
PAH	Percentage^a^	95% CI	p-value	Percentage^a^	95%CI	p-value
All	−9.78	−15.48 to −3.70	0.003	0.41	−5.64 to 6.86	0.9
Volatile	−7.30	−13.71 to −0.42	0.04	−1.65	−8.77 to 6.02	0.67
Non-volatile	−9.85	−15.16 to −4.2	0.002	1.14	−4.69 to 7.32	0.71
Benzo*(a)*pyrene	−7.18	−11.82 to −2.3	0.007	0.086	−5.85 to 6.39	0.98

a Percentage was calculated for each doubling in PAHs exposure (based on a model with log PAH and log mtDNA-content).

#### Biomarkers of oxidative DNA damage

No significant associations were found between plasma isoprostane levels and 8-deoxyhydroxyguanosine and PAHs in indoor dust (table 5).

**Table 5:** Estimated change (95% CI) in plasma isoprostane and Urinary 8-hydroxydeoxyguanosine in association with PAH exposure and mitochondrial DNA (mtDNA) content.

		Winter			Summer		
		Percentage^a^	95% CI	p-value	Percentage^a^	95%CI	p-value
Plasma Isoprostane							
	All PAHs	14.56	−9.04 to 38.18	0.23	−2.58	−36.06 to 30.90	0.88
	Volatile PAHs	14.99	−9.68 to 39.67	0.24	−34.51	−73.7 to 4.68	0.09
	Non-volatile PAHs	13.17	−9.17 to 35.51	0.25	10.23	−21.55 to 42	0.53
Urinary 8-hydroxydeoxyguanosine							
	All PAHs	1.53	−4.92 to 7.98	0.64	0.09	−5.98 to 6.17	0.98
	Volatile PAHs	6.11	−0.45 to 12.68	0.07	1.38	−5.88 to 8.64	0.71
	Non-volatile PAHs	1.13	−4.94 to 7.20	0.72	0.0001	−5.83 to 5.83	0.99

a Percentage was calculated for each doubling in isoprostane and 8-hydroxydeoxyguanosine (based on a model with isoprostane, 8-hydroxydeoxyguanosine and log PAH).

Adjusted for gender, age, BMI and the consumption of grilled meat or fish. 8-hydroxydeoxyguanosine was additionally adjusted for urinary creatinine levels.

Adjusted for gender, age, BMI and the consumption of grilled meat or fish.

### Cell Culture

**Figure 3:**
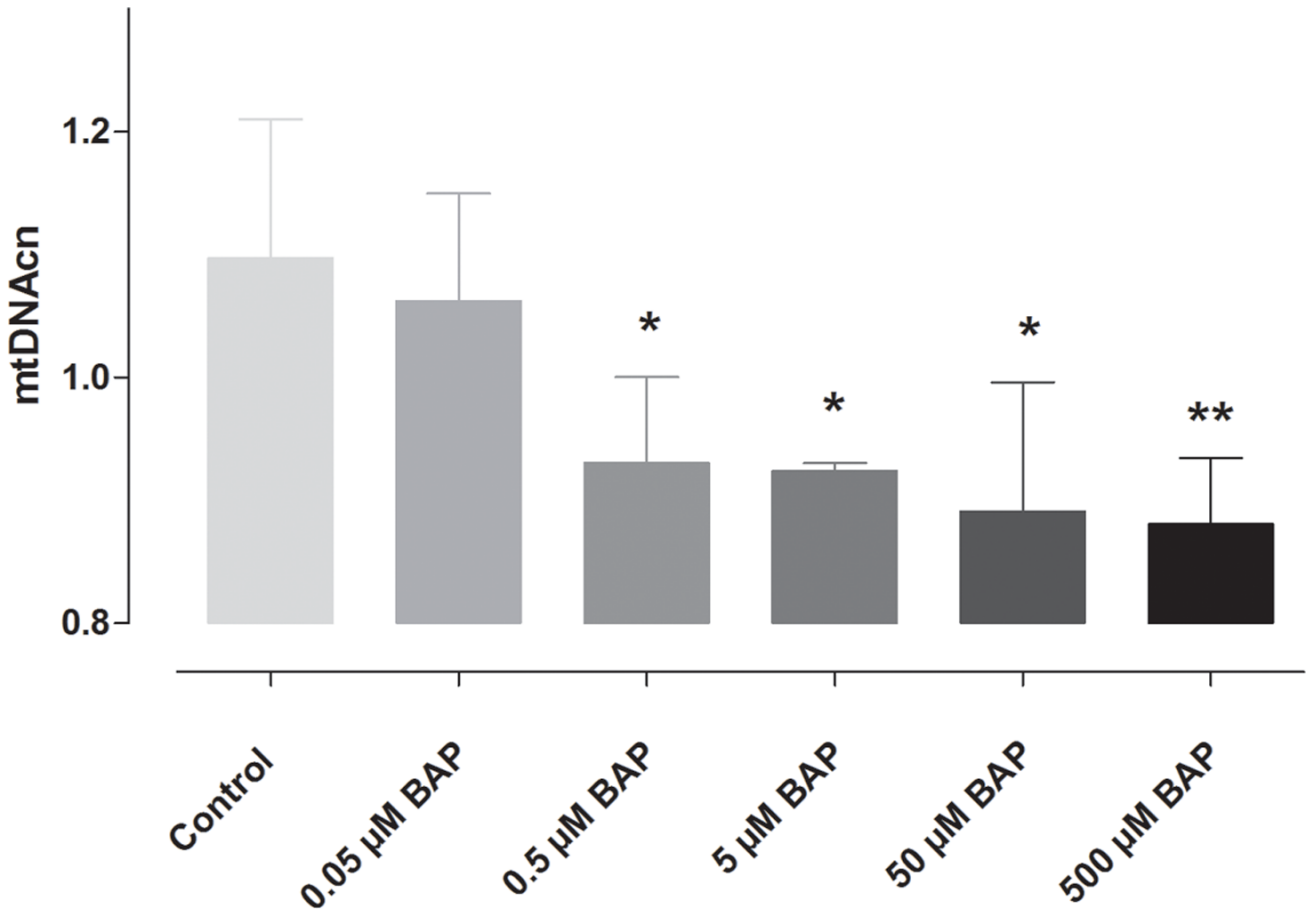
Mean mitochondrial DNA content in response to benzo*(a)*pyrene. Mean mitochondrial DNA (mtDNA) content of human TK6 cells exposed to 0; 0.05; 0.5; 5; 50 and 500 µM benzo*(a)*pyrene (BAP). Data are presented as mean ± SD; n = 3. *p<0.05 vs control (0 µM BAP); **p<0.01 vs. control (Analysis of variance: Kruskall-Wallis). Jonckheere-Terpstra test showed a significant (p = 0.0011) decrease over the exposure range.

Human TK6 cells, exposed for 24 h to different concentrations of benzo*(a)*pyrene (0 to 500 µM benzo*(a)*pyrene) showed a significant dose-dependent decrease in mitochondrial DNA content. The concentration of 0.5 µM and higher showed significant decreases in comparison with the control group and the cells exposed to the lowest concentration of 0.05 µM benzo*(a)*pyrene (figure 3). The Jonckheere-Terpstra test showed a significant decrease over the different exposures (p = 0.0011). In S9 treated cells no decrease in mitochondrial DNA content was observed over the exposure range. TK6 cells viability and number of dead and living cells are given in table 6. Cell viability was comparable for all conditions, however, since the total number of living cells decreased with higher benzo*(a)*pyrene exposure, exposure to benzo*(a)*pyrene does not cause acute cytotoxicity but suppresses cell growth.

**Table 6:** TK6 cells viability and number of dead and living cells per exposure condition to benzo*(a)*pyrene (BAP).

	Cell viability (%)	Number of cells (×10^6/^ml)	Number of dead cells (×10^4^/ml)
Control	96	1.37	5.66
0.05 µM BAP	96	1.27	5.33
0.5 µM BAP	97	1.00	4.33
5 µM BAP	96	0.81	3.00
50 µM BAP	97	0.90	3.00
500 µM BAP	94	0.91	6.33

## Discussion

The mitochondrial DNA content correlates with the size and number of mitochondria, which have been shown to change under different energy demands, as well as different pathological conditions [4]. Experimental studies demonstrated that any genetic manipulation resulting in significantly decreased mitochondrial DNA content accelerates the ageing process and causes age-related disorders [21]. Therefore, mitochondrial DNA content might be an important and relevant target to study the effects of environmental exposures including PAHs. The key finding of our study is that mitochondrial DNA content is inversely associated with indoor exposure to PAHs in dust, including the group 1 carcinogen benzo*(a)*pyrene, in winter. This association was independent of gender, age, BMI and the consumption of grilled meat or fish. These findings were experimentally established in human TK6 cells, where mitochondrial DNA content also decreased in function of the benzo*(a)*pyrene concentration.

Our results are in line with a recent study on smoking [22]. In this study, a decrease in mitochondrial DNA content was observed in the lungs of smokers, which was attributed to the induced oxidative stress. Cigarette smoke contains many compounds, including PAHs (benzo*(a)*pyrene). In contrast to our results, others found that blood mitochondrial DNA content was increased in various occupational groups exposed to low benzene levels [23]. It has been suggested that the increased oxidative stress, caused by exposure to PAHs, has a dual influence on mitochondrial DNA content. Mild stress can stimulate mitochondrial DNA production and the number of mitochondria to fulfill in the respiratory needs of the cell and, as such, the cell will survive. But excessive oxidative stress may result in decreased or no synthesis of mitochondrial DNA due to the increasing abundance of defect mitochondria, eventually leading to cell senescence or cell death [8]. However, at the investigated concentrations no significant associations between PAHs in indoor dust and indicators of oxidative stress at the lipid level and nuclear DNA, as exemplified by plasma isoprostane level and urinary-8-hydroxydeoxyguanosine, respectively, were found.

Changes in the ratio between mitochondrial DNA content and nuclear DNA may be related to the development of multiple forms of disease. To date, many studies reported increases or decreases in mitochondrial DNA content in response to endogenous or exogenous factors. Decreased mitochondrial DNA content has been shown in type II diabetes [24], [25], [26], soft cell sarcoma [27], ovarian cancer [28], breast cancer [29], [30], gastric cancer [31], hepatocellular carcinoma [32] and renal cell carcinoma [33]. Whether mitochondrial DNA content depletion has a role in tumorogenesis, is still under investigation, but is was demonstrated that extensive oxidative stress in cancer cells can cause changes in mitochondrial DNA content. This leads to alterations in mitochondrial gene expression and causes a deficiency in oxidative phosphorylation (OXPHOS) [34]. A diminished OXPHOS activity was also demonstrated in aged tissues [35]. Mitochondrial mutations and simultaneous decreases in mitochondrial DNA content can ultimately lead to a detrimental cycle of further damage of the mitochondrial DNA but also genotoxic damage with rapid erosion and damage of telomeres [21]. As ageing is a complex process involving defects in various cellular components, we hypothesize that the changes observed on mitochondrial DNA content might be a relevant mechanism for cellular ageing by PAHs. Future studies on the link between PAH exposure and mitochondrial DNA content in association with effects observed in the nucleolus, such as telomere erosion, are necessary to elucidate the potential ageing pathways induced by exposure to PAHs.

We found no associations between indoor PAH exposure and mitochondrial DNA content in summer. This might be explained by several factors. PAH levels in the indoor environment during summer were lower. Residences with stove or open fire, tended (not significantly, probably due to the low number) to show a higher median indoor concentration of benzo*(a)*pyrene, non-volatile PAHs and volatile PAHs in winter. Also, during summer, people spend more time outdoors and their residences are in general more ventilated than in winter. Therefore, house dust in summer might be a less relevant PAH exposure marker.

Although our results were consistent after multiple adjustments, we cannot exclude that our associations obtained were due to residual confounding or were caused by some unknown factor that is associated with both mitochondrial function and exposure to PAHs. A clear limitation of this study is its small sample size. Since we analyzed DNA from whole blood we are capturing a mixture of leukocytes and the associations may be due to differences in white blood cell subpopulations. Further, changes in mitochondrial DNA content in human blood cells could also be attributed to platelet variation [36]. Platelet contamination increases mitochondrial DNA without an augmentation in nuclear DNA and affects mitochondrial DNA content [37]. However, in a previous study by Janssen et al. 2012 [38], mitochondrial DNA did not correlate with blood platelets, neutrophils, white blood cells or white blood cell/platelet ratio. A clear strength is that our observational data in humans are confirmed by observations in cell cultures. We observed a dose dependent decrease in the mitochondrial DNA content of human lymphoblastoid TK 6 cells over a wide range of benzo*(a)*pyrene exposures.

In conclusion, PAH exposure in winter is associated with mitochondrial damage as exemplified by mitochondrial DNA content. Changes in mitochondrial DNA content might be an early target of PAH exposure. The potential health consequences of decreased mitochondrial DNA content and the role of PAHs in the ageing process must be further elucidated.

## Methods

### Population Study

#### Ethics statement

Written informed consent was provided by all study participants in accordance with procedures approved by the Ethical Committee of the University of Antwerp (Reference nr. UA A09 22).

#### Subjects

We recruited two household members of 24 families. Only non-smokers, living in a smoke-free house and living for at least one year at their current residence, were included. The total population included 46 participants. A self-administered questionnaire was used to collect information on socio-economic status, lifestyle, general health, use of medication and the presence of risk factors. There were two sampling periods, one in winter (18 February– 2 March 2010) and one in spring/summer (5 June– 25 June 2010). In the period of dust sampling, blood- and urine samples were collected. Each participant was asked to complete a form with questions concerning diet and exposure during the 15 hours, prior to sampling.

#### Exposure measurement

The inhabitants of the residences collected sedimented house dust during 3 weeks using a vacuum cleaner. Also, blood and urine samples were collected within this period. The fine dust (ca. <100 µg) in between two paper layers of the dust bag was extracted using soxhlet extraction. To eliminate interference of fats from food leftovers and products of biological origin, the PAH fraction was separated from the fat fraction by gel permeation chromatography. Additional clean-up was performed using a combined silica/alumina column and the final sample was analysed with gas chromatography-mass spectrometry. Eight PAHs, typically considered as possible carcinogens, are described as non-volatile PAHs (benzo*(a*)anthracene, chrysene, benzo*(b)*fluoranthene, benzo*(k)*fluoranthene, benzo*(a)*pyrene, dibenzo*(a,h)*anthracene, benzo*(ghi)*perylene, *indeno(1,2,3-cd)*pyrene) [39], [40] and were measured in this study. Acenaphthylene, acenapthene, fluorine, phenanthrene, anthracene and the possible carcinogenic compound naphthalene, are specified as volatile PAHs. Fluoranthene and pyrene were also measured.

#### Biomarkers of oxidative damage

For analysis of plasma 15-F2T-isoprostane, a marker of lipid peroxidation, 500 µl plasma was collected in dark tubes containing 2 µl butylhydroxytoluene (5 ng/ml 100% ethanol). Plasma 15F2T-isoprostane (pg/ml) was determined using an enzyme immuno-assay kit (Cayman Chemicals, Ann Arbor, MI, USA), according to the manufacturer’s specifications. Urinary 8-deoxyhydroxyguanosine, a reflection of oxidative DNA damage, was measured with the New 8-OHdG Check from the Japan Institute for the control of aging (Gentaur, Kampenhout, BE).

### Cell Culture Experiment

Benzo(a)pyrene was tested in the human lymphoblastoid cell line TK6. TK6 cells were purchased from the European Collection of Cell Cultures (ECACC, Wiltshire, UK) and maintained in RPMI 1640 medium (Invitrogen, Merelbeke, BE) containing 10% heat-inactivated fetal calf serum, 100 U/ml penicillin, 100 µg/ml streptomycin and 2 mM l-glutamine at 5% CO_2_ and 37°C. Prior to exposure, TK6 cells were seeded into 48 well-plate at a concentration of 0.5×10^6^ cells/well.

Benzo*(a)*pyrene (Sigma NV/SA, Bornem, BE) was dissolved in dimethyl sulfoxide (Sigma NV/SA, Bornem, BE). The solvent concentration (v/v) of the final culture volume was 1%. A mixture of S9 (1% v/v, Celsis, Neuss, GER) from human liver was added to the culture in half of the experiments. Cells were divided into 5 treatment groups (0.05, 0.5, 5, 50 and 500 µM benzo(a)pyrene), in either the presence or absence of S9 mix. An exogenous NADPH-regenerating system (BD Biosciences, Erembodemgem BE) required by liver S9 for phase I oxidation was included in the experiments. Two solvent control groups (control S9−, control S9+) were also included. Because of the potential cytotoxicity of S9 preparations for cultured mammalian cells [41] a short term treatment (3 h) in the presence and absence of S9 was followed by removal of the test substance and a growth period of 21 h. Cells were exposed to benzo*(a)*pyrene in triplicates.

At the end of the exposure period, the cytotoxic response was evaluated with lactate dehydrogenase (LDH) activity assay as described previously [42]. The LDH measurement assesses membrane damage and is, therefore, indicative for cell death. We also counted the cells and assessed proportions of living and dead cells using a Countess™ Automated Cell Counter (Invitrogen, Carlsbad, CA).

### Measurement of Mitochondrial DNA Content

For the population study, we collected whole blood in an heparin-coated vacutainer (BD, Franklin Lakes, NJ, USA) and total DNA was extracted from whole blood using a QIAamp DNA blood Maxi kit (QIAgen, Hilden, GER) following the manufacturer’s instructions. For the TK6 cells, DNA was extracted with the QIAamp DNA mini kit (Qiagen, Hilden, GER). The concentration of extracted DNA was measured at 260 nm with the Nanodrop spectrophotometer (ND-1000, Isogen Life Science, De Meern, NE). Both DNA yield (ng/µl), which averaged 255 ng/µl for the blood samples and 82 ng/µl for the TK6 cells, and purity ratios A260/280 (range: 1.63–2.10) and A260/230 (range: 1.28–2.51) were determined. Extracted DNA was stored at −20°C until further use.

Relative mitochondrial DNA content was estimated using a quantitative real-time PCR (qPCR) assay by determining the ratio of the ND-1 mitochondrial gene to two nuclear reference genes (β-act and 36B4). Extracted genomic DNA was diluted to a final concentration of 5 ng/µl in RNase free water, prior to the qPCR runs. A 10 µl PCR reaction contained Fast SYBR® Green I dye 2× (Applied biosystems, Lennik, BE) mastermix, forward (10 µM) and reverse (10 µM) primer and 12.5 µg DNA. Primer details and efficiency are shown in table 7. All PCR-reactions were performed on a 7900HT Fast Real-Time PCR System (Applied Biosystems, Foster City, CA, USA). The thermal cycling profile was similar for mitochondrial DNA and nuclear DNA and consisted of following steps: 20 s at 95°C to activate the AmpliTaq Gold® DNA-polymerase, followed by 40 cycles of 1 s at 95°C for denaturation and 20 s at 60°C for annealing/extension. Each run was completed by a melting curve analysis to confirm the amplification specificity and absence of non-specific PCR products. Each PCR-plate contained four inter-run calibrators and two no-template controls. After thermal cycling, raw data were collected and processed. The range of the C_q_ values of the used genes and the no-template controls are given in table 7. C_q_-values of the mitochondrial gene were normalized relative to the two reference genes using the qBase software (Biogazelle, Zwijnaarde, BE). The program uses modified software from the classic comparative C_T_ method (ΔΔ C_T_) that takes into account multiple reference genes and uses inter-run calibration algorithms to correct for run-to-run differences [43].

**Table 7:** Characteristics of selected genes for qPCR.

Gene symbol	ND-1	β-act	36B4
Nuclear/Mitochondrial	Mitochondrial	Nuclear	Nuclear
Accession number	NC_012920.1	NM_001101.3	NM_001002.3
Amplicon length (bp)	115	102	84
Forward 5′–3′	ATGGCCAACCTCCTACTCCT	ACTCTTCCAGCCTTCCTTCC	GGAATGTGGGCTTTGTGTTC
Reverse 5′–3′	CTACAACGTTGGGGCCTTT	GGCAGGACTTAGCTTCCACA	CCCAATTGTCCCCTTACCTT
Primer efficiency (%)*	99.3%–104%	92%–96.8%	98%–100.7%
C_q_ range	16.36–18.75	23.47–25.45	23.21–24.93
Non template control range	32.42 - Undetermined	36.12 - Undetermined	35.92 - Undetermined

* Primer efficiency was determined in two different experiments.

Mitochondrial encoded NADH dehydrogenase 1 (ND-1; Beta actin (β-actin); Acidic ribosomal phosphoprotein P0 (36B4).

### Statistical Analysis

Statistical analyses were conducted using the SAS statistical package, version 9.2 (SAS Institute, Cary, NC, USA). The association between mitochondrial DNA content and PAH exposure was examined by mixed models using the MIXED procedure. Both mitochondrial DNA content and PAH exposure were log transformed and treated as continuous variables. Individuals nested within households were treated as a random factor and were included in the model to control for correlation between repeated observations at the level of the individual as well as the household. Models were adjusted for the following fixed effects: gender, age, body mass index and the consumption of grilled meat or fish during the last three days. Because the inclusion of an interaction term between season and the exposure revealed significant effect modification, we stratified analyses by season.

Cell culture data were analyzed using the non-parametric Kruskall-Wallis test. To study the trend over the exposure range, we used the Jonckheere-Terpstra test.
